# Low PAPPA and Its Association with Adverse Pregnancy Outcomes in Twin Pregnancies: A Systematic Review of the Literature and Meta-Analysis

**DOI:** 10.3390/jcm13226637

**Published:** 2024-11-05

**Authors:** Ioakeim Sapantzoglou, Maria Giourga, Afroditi Maria Kontopoulou, Vasileios Pergialiotis, Maria Anastasia Daskalaki, Panagiotis Antsaklis, Marianna Theodora, Nikolaos Thomakos, George Daskalakis

**Affiliations:** 1st Department of Obstetrics and Gynecology, Alexandra Hospital, National and Kapodistrian University of Athens, Vasilissis Sofias 80 Aven., 11528 Athens, Greece; kimsap1990@hotmail.com (I.S.); giourga.m@gmail.com (M.G.); ama_kontopoulou@yahoo.com (A.M.K.); pergialiotis@hotmail.com (V.P.); anastasia.daskalaki00@gmail.com (M.A.D.); panosant@gmail.com (P.A.); martheodr@gmail.com (M.T.); thomakir@hotmail.com (N.T.)

**Keywords:** PAPP-A, biochemical analytes, twin pregnancy, adverse outcomes, twin gestation, serum biomarkers, perinatal outcomes

## Abstract

**Background**: It is well established in the literature that pregnancy-associated plasma protein-A (PAPP-A) is linked to several adverse pregnancy outcomes, including pre-eclampsia (PE), fetal growth restriction (FGR), and preterm birth (PTB) in singleton pregnancies. However, data regarding such an association in twin pregnancies are lacking. The primary goal of this systematic review and meta-analysis was to assess the potential value of low PAPP-A levels in the prediction of the subsequent development of hypertensive disorders of pregnancy (HDPs), PTB, and small for gestational age (SGA)/FGR fetuses in twin pregnancies and investigate its association with the development of gestational diabetes, intrauterine death (IUD) of at least one twin, and birth weight discordance (BWD) among the fetuses. **Methods**: Medline, Scopus, CENTRAL, Clinicaltrials.gov, and Google Scholar databases were systematically searched from inception until 31 July 2024. All observational studies reporting low PAPP-A levels after the performance of the first-trimester combined test as part of the screening for chromosomal abnormalities with reported adverse pregnancy outcomes were included. **Results**: The current systematic review encompassed a total of 11 studies (among which 6 were included in the current meta-analysis) that enrolled a total of 3741 patients. Low PAPP-A levels were not associated with HDPs (OR 1.25, 95% CI 0.78, 2.02, I-square test: 13%). Low PAPP-A levels were positively associated with both the development of preterm birth prior to 32 (OR 2.85, 95% CI 1.70, 4.77, I-square test: 0%) and 34 weeks of gestational age (OR 2.09, 95% CI 1.34, 3.28, I-square test: 0%). Furthermore, low PAPP-A levels were positively associated with SGA/FGR (OR 1.58, 95% CI 1.04, 2.41, I-square test: 0%). Prediction intervals indicated that the sample size that was used did not suffice to support these findings in future studies. **Conclusions**: Our study indicated that low PAPP-A levels are correlated with an increased incidence of adverse perinatal outcomes in twin pregnancies. Identifying women at elevated risk for such adversities in twin pregnancies may facilitate appropriate management and potential interventions, but additional studies are required to identify the underlying mechanism linking PAPP-A with those obstetrical complications.

## 1. Introduction

The development and improvements of assisted reproductive technology (ART) have given the opportunity of conception to a large proportion of subfertile women, and such progress has resulted in a considerable rise in the occurrence of multiple pregnancies with current research, indicating that this increase ranges from 15% to 38% [1,2]. At the same time, however, twin pregnancies give rise to an increased likelihood of significant obstetrical adversities, such as preterm birth (PTB), fetal growth restriction (FGR), and hypertensive disorders of pregnancy (HDPs), leading to significant perinatal morbidity and mortality [3,4]. It is well established in the current literature that pregnancy-associated plasma protein-A (PAPP-A), which is a maternal serum biomarker used in the first-trimester combined screening test for chromosomal abnormalities and is derived from the syncytial trophoblast, is linked to a number of adverse pregnancy outcomes, including pre-eclampsia (PE), FGR, and PTB in singleton pregnancies [5,6].

Nevertheless, there is a dearth of comprehensive data on first-trimester biomarkers in twin pregnancies and their association with the subsequent development of adverse pregnancy outcomes with several prior studies having presented contradictory findings [7,8]. As such, the primary goal of this systematic review and meta-analysis was to assess and investigate the potential value of low PAPP-A levels in the prediction of subsequent HDPs, PTB, and SGA/FGR in twin pregnancies and its association with the development of GDM, intrauterine death (IUD) of at least one twin, and birth weight discordance (BWD) among the fetuses, hoping to determine the clinical relevance of this biochemical marker and the potential necessity for additional investigation of its utility in complications other than the detection of common aneuploidies.

## 2. Materials and Methods

This systematic review and meta-analysis was designed according to the Preferred Reporting Items for Systematic Reviews and Meta-Analyses (PRISMA) guidelines, as well as according to the MOOSE Guidelines for Meta-Analyses and Systematic Reviews of Observational Studies [9]. This review was registered in the PROSPERO international database for systematic reviews (reference: CRD42024589643).

### 2.1. Eligibility Criteria

The present systematic review included all observational studies (prospective/retrospective cohort, case–control, nested case–control, and cross-sectional including both spontaneous and in vitro fertilization (IVF) pregnancies) that reported low PAPP-A levels after the performance of the first-trimester combined test as part of the screening for chromosomal abnormalities and their association with adverse pregnancy outcomes, namely the subsequent development of hypertensive disorders of pregnancy, preterm birth prior to 34 and 32 weeks of gestational age, gestational diabetes, the detection of small for gestational age fetuses or growth-restricted fetuses, discordance of fetal growth among the two fetuses, and intrauterine death of at least one twin. No laboratory assay restrictions were applied. Case reports, small case series, letters to the editor, animal studies, and review articles were not included. Conference proceedings and abstracts were also planned to be excluded, as they lack important information that is necessary for the assessment of study limitations and quality of evidence.

### 2.2. Information Sources and Search Strategy

**Figure 1:**
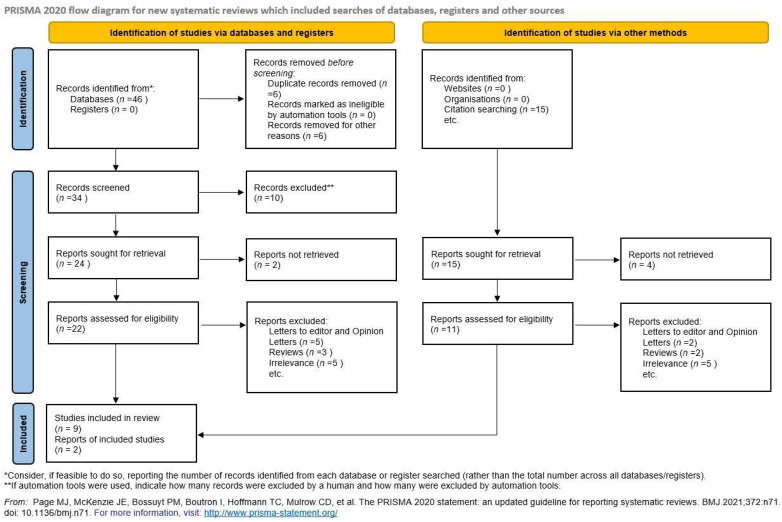
Search strategy [10].

The Medline (1966–2024), Scopus (2004–2024), Clinicaltrials.gov (2008–2024), EMBASE (1980–2024), Cochrane Central Register of Controlled Trials CENTRAL (1999–2024), and Google Scholar (2004–2024) databases were used in our primary search, along with the reference lists of electronically retrieved full-text papers. The date of our last search was set at 31 July 2024. Our search strategy included the text words ‘PAPP-A’, ‘biochemical analytes, ‘twin pregnancy’, ‘adverse outcomes’, ‘twin gestation’, ‘serum biomarkers’, and ‘perinatal outcomes’ and is briefly presented in Figure 1. The main search algorithm was as follows: (‘PAPP-A’ OR ‘biochemical analytes’ OR ‘serum biomarkers’) AND (‘twin pregnancy’ OR ‘twin gestation’) AND (‘adverse outcomes’ OR ‘perinatal outcomes’). The search identified 61 potentially relevant studies, but 50 were excluded because they were non-relevant articles, reviews, opinion letters, or letters to the editor, or we were unable to retrieve the available data after contacting the authors. Thus, in total, only 11 peer-reviewed papers were considered for inclusion in our systematic review, among which 6 were included in the current meta-analysis. A meta-analysis regarding the potential association between low PAPP-A levels and the subsequent development of GDM, birth weight discordance, and IUD was omitted due to limited data.

### 2.3. Study Selection

The study selection process involved three consecutive stages. First, the titles and abstracts of all electronic papers were screened to assess their potential eligibility. Subsequently, all articles that met or were presumed to meet the eligibility criteria were retrieved as full texts. Finally, all observational (both prospective and retrospective) twin studies reporting the PAPP-A levels as well as data regarding the incidence of adverse pregnancy outcomes (HDPs, GDM, PTB, small for gestational age (SGA)/FGR, IUD, and BWD) were deemed eligible. Study selection was performed by two authors independently, while any potential discrepancies were resolved through their consensus (Figure 1).

### 2.4. Data Collection

The following data were extracted from each included study: name of first author, year of publication, study design, study center, recruitment period, timing of assessment, inclusion and exclusion criteria, the cut-off that defined low PAPP-A (either in multiples of median (MoM) or centiles), number of patients, maternal age, race, body mass index (BMI) at testing, BMI at birth, presence of prior diabetes mellitus, parity, smoking status, presence of prior chronic hypertension, and birth weight. When important data were missing, attempts were made to contact corresponding authors. Data extraction was performed by three authors, and any possible disagreements were resolved through consensus or by discussion with all authors. The demographic and methodological characteristics of the included studies are depicted in Table 1 and Table 2, respectively.

### 2.5. Quality Assessment

**Figure 2:**
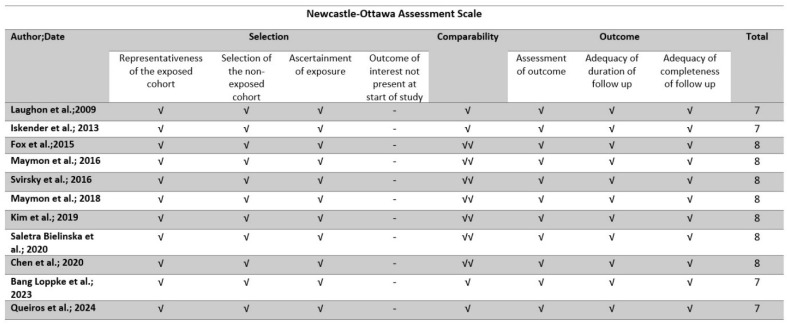
Newcastle–Ottawa scale (NOS) quality assessment of the included studies [7,8,9,10,11,12,13,14,15,16,17]. √: the selected quality item was evaluated and found present in the study (selection and outcome categories), √: the study groups were controlled for one important factor (gestational age) (comparability category), √√: the study groups were controlled for two important factors (gestational age and maternal weight) (comparability category).

The methodological quality of all the included studies was evaluated using the Newcastle–Ottawa scale (NOS) tool, which is a widely utilized instrument for evaluating the methodological quality of non-randomized studies that are incorporated in systematic reviews and/or meta-analyses [20]. The tool assesses each study based on eight criteria, which are divided into three categories: the selection of study groups; the comparability of the groups, which was based on the maternal weight and the gestational age; and the determination of either the exposure or outcome of interest for case–control or cohort studies, respectively. Stars were assigned to each quality item as a means of providing a rapid visual evaluation. Stars were allocated in a manner that granted the highest caliber studies a maximum of nine stars. The tool was implemented by two authors independently, and any discrepancies were resolved through discussion with a third author. Overall, the risk of bias was assessed to be good, fair, or poor (Figure 2).

### 2.6. Data Synthesis

Statistical meta-analysis was performed with RStudio using the meta function accessed on 30 July 2024 (RStudio Team (2015). RStudio: Integrated Development for R. RStudio, Inc., Boston, MA, URL: http://www.rstudio.com/) [21]. Statistical heterogeneity was not considered during the evaluation of the appropriate model (fixed effects or random effects) of statistical analysis as the considerable methodological heterogeneity (Table 1) did not permit the assumption of comparable effect sizes among the studies included in the meta-analysis [22]. Confidence intervals were set at 95%. We calculated pooled odds ratios (ORs) as well as 95% confidence intervals (CIs) with the Hartung–Knapp–Sidik–Jonkman instead of the traditional Dersimonian–Laird random-effect model (REM) analysis. We opted to use this model as recent research indicates its superiority to the Dersimonian–Laird model in terms of accounting for the heterogeneity of included observational studies that are expected to differ considerably in their methodology.

### 2.7. Prediction Intervals

Prediction intervals (PIs) were also calculated, using the meta function in RStudio, to evaluate the estimated effect expected in future studies in the field. The estimation of prediction intervals considers the inter-study variation in the results and expresses the existing heterogeneity at the same scale as the examined outcome.

## 3. Results

Our search identified 61 potentially relevant studies, but 50 were excluded after reviewing the titles and the abstracts and removing non-relevant articles, case reports, opinion letters, reviews, and letters to the editor. Overall, 11 studies were included in the present systematic review (6 retrospective cohort studies [8,11,12,13,16,19], 4 prospective cohort [7,14,15,17] studies, and 1 register-based national cohort study [18]) that enrolled a total of 3741 patients. The search strategy and the quality assessment are briefly presented in Figure 1 and Figure 2, respectively. The demographic characteristics of the patients included and the methodological characteristics of the included studies are summarized in Table 1 and Table 2, respectively. Eight of the included studies [7,8,12,13,14,16,17,19] investigated the association between low PAPP-A levels and the subsequent development of hypertensive disorders of pregnancy (PE or PIH); three of them [8,11,19] investigated the association between low PAPP-A levels and the development of PTB prior to 32 weeks of gestation; three studies [8,12,19] investigated the association between low PAPP-A levels and the development of PTB prior to 34 weeks; three studies investigated the subsequent development of GDM [8,12,15]; four studies assessed the potential association with the development of SGA/FGR [8,12,13,19], while two of the included studies investigated the correlation between low PAPP-A levels and IUD [8,13] and three with discordance in fetal growth [8,12,18].

The gestational age of the blood sampling for the investigation of PAPP-A was limited in the first trimester and ranged from 10 to 14 gestational weeks among the studies included.

The cut-off that defined low PAPP-A varied among the included studies, with three studies adopting the 10th centile of their measurements, one the 25th centile, and one using values lower than 0.42 MoMs, while the rest did not specify their cut-off.

The definition of PE varied across the studies, as four studies adopted the criteria of the International Society for the Study of Hypertension in Pregnancy (ISSHP), as issued by Sibai [23] on behalf of the American Society of Maternal and Fetal Medicine; one defined PE in accordance to the American College of Obstetricians and Gynecologists Practice Bulletin Number 202 [24]; and one adopted the revised criteria of the ISSHP [25], while two did not specify the diagnostic criteria used. The definition of GDM also varied among studies, as one study adopted the 2013 American College of Obstetricians and Gynecologists Practice guidelines [26], and one adopted the national guidelines [27], while one study did not specify the criteria used. Preterm delivery was defined as delivery prior to 34 or 32 weeks of gestational age. Small for gestational age was defined as a birth weight below the 10th centile for the given gestational age, and FGR was defined as Doppler abnormalities in an SGA fetus. IUD was diagnosed by the demise of a fetus after 22 weeks of gestational age. In terms of growth discordance, two studies included twin pregnancies with a birth weight discordance of ≥25%, while one defined it as a birth weight discordance of ≥20%.

### Synthesis of Results

**Figure 3:**
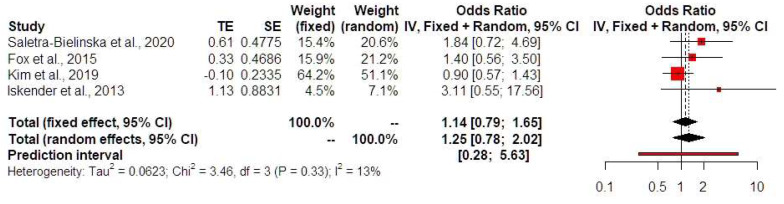
Forest plots of odds ratios of the subsequent development of hypertensive disorders of pregnancy in twin pregnancies with low PAPP-A levels with 95% confidence intervals (CIs) and weighted pooled summary statistics using a bivariate random-effect model [8,12,13,16]. Forest plot analysis: Vertical line = “no difference” point between the two groups. Red squares = Odds ratios of individual studies; Diamond = pooled odds ratios and 95% CI for all studies; Horizontal black lines = 95% CI; Horizontal red line = prediction intervals. Abbreviations: SE: standard error, CI: confidence interval.

**Figure 4:**
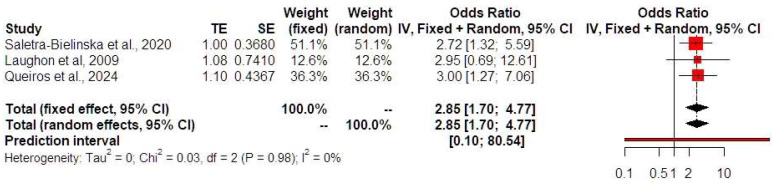
Forest plots of odds ratios of the subsequent development of preterm birth prior to 32 weeks of gestational age in twin pregnancies with low PAPP-A levels with 95% confidence intervals (CIs) and weighted pooled summary statistics using a bivariate random-effect model [8,11,19]. Forest plot analysis: Vertical line = “no difference” point between the two groups. Red squares = Odds ratios of individual studies; Diamond = pooled odds ratios and 95% CI for all studies; Horizontal black lines = 95% CI; Horizontal red line = prediction intervals. Abbreviations: SE: standard error, CI: confidence interval.

**Figure 5:**
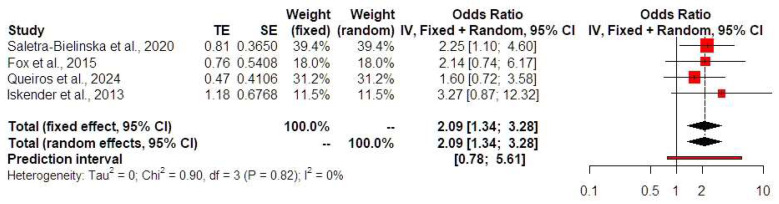
Forest plots of odds ratios of the subsequent development of preterm birth prior to 34 weeks of gestational age in twin pregnancies with low PAPP-A levels with 95% confidence intervals (CIs) and weighted pooled summary statistics using a bivariate random-effect model [8,12,13,19]. Forest plot analysis: Vertical line = “no difference” point between the two groups. Red squares = Odds ratios of individual studies; Diamond = pooled odds ratios and 95% CI for all studies; Horizontal black lines = 95% CI; Horizontal red line = prediction intervals. Abbreviations: SE: standard error, CI: confidence interval.

**Figure 6:**
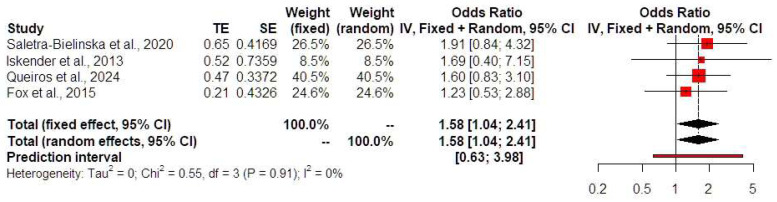
Forest plots of odds ratios of the subsequent development of SGA/FGR in twin pregnancies with low PAPP-A levels with 95% confidence intervals (CIs) and weighted pooled summary statistics using a bivariate random-effect model [8,12,13,19]. Forest plot analysis: Vertical line = “no difference” point between the two groups. Red squares = Odds ratios of individual studies; Diamond = pooled odds ratios and 95% CI for all studies; Horizontal black lines = 95% CI; Horizontal red line = prediction intervals. Abbreviations: SE: standard error, CI: confidence interval.

Low PAPP-A levels were not associated with HDPs (OR 1.25, 95% CI 0.78, 2.02, I-square test: 13%) (Figure 3). Prediction intervals indicated that the sample size that was used did not suffice to support these findings in future studies. Low PAPP-A levels were positively associated with both the development of preterm birth prior to 32 (OR 2.85, 95% CI 1.70, 4.77, I-square test: 0%) (Figure 4) and 34 weeks of gestational age (OR 2.09, 95% CI 1.34, 3.28, I-square test: 0%) (Figure 5). Prediction intervals indicated that the sample size used did not suffice to support these findings in future studies. Furthermore, low PAPP-A levels were positively associated with SGA/FGR (OR 1.58, 95% CI 1.04, 2.41, I-square test: 0%) (Figure 6). Prediction intervals indicated that the sample size used did not suffice to support these findings in future studies.

In terms of the rest of the investigated outcomes (GDM, IUD, discordant fetal growth), a summary of their association with PAPP-A levels is briefly presented in Table 3. Three studies investigated the subsequent development of GDM [8,12,15], with one demonstrating a nonsignificant association between low levels of PAPP-A and GDM, one demonstrating a statistically significant correlation, while one study revealed an association between high levels of PAPP-A with the subsequent development of GDM. Two of the included studies investigated the correlation between low PAPP-A levels and IUD [8,13], with both revealing a nonsignificant association, and three assessed the association with discordance in fetal growth [8,12,18], with all of them demonstrating a lack of association.

## 4. Discussion

To the best of our knowledge, this is the first systematic review and meta-analysis on the possible association of low PAPP-A levels and the subsequent occurrence of adverse perinatal outcomes in twin pregnancies. The main findings of our study revealed that low PAPP-A levels are associated with an increased rate of adverse perinatal outcomes in twin pregnancies, namely the development of subsequent PTB prior to 32 and 34 weeks, as well as SGA/FGR, while the collected data did not demonstrate a statistically significant correlation with the subsequent development of hypertensive disorders of pregnancy. As such, low PAPP-A levels have the potential to be used as predictive biomarkers for adverse pregnancy outcomes, and understanding the association between low PAPP-A levels and these outcomes could lead to the early identification of pregnancies at risk, enabling obstetricians to appropriate intervention and therefore enhance maternal–fetal outcomes.

### 4.1. Low PAPP-A and PTB

It is well established that reduced levels of PAPP-A are correlated with impaired placental function and preterm delivery in singleton pregnancies [28], possibly through a mechanism according to which low PAPP-A results in the downregulation of IGF 2 availability, leading to defective trophoblast invasion into maternal decidua and aberrant placentation in early pregnancy. Consequently, the compromised placentation and placental ischemia resulting from reduced IGF bioavailability during early gestation may influence the incidence of preterm delivery; however, the precise mechanism remains unclear [29].

Numerous authors have evaluated the correlation between first-trimester aneuploidy biochemical markers, including PAPP-A, and perinatal outcomes in twins, yielding perplexing results. While the studies of Rosner et al. [13] and Saletra-Bielinska et al. [8] revealed a significantly higher risk of preterm delivery in twin pregnancies affected by low PAPP-A levels, such an association could not be demonstrated by other authors [11,12]. This was mainly attributed to the small sample size of the studies by Laughon et al. and Iskender et al., as they included only 70 and 104 twin pregnancies in their analysis, respectively, with the results of the study by Laughon et al. revealing that delivery prior to 32 weeks of gestation occurred about three times more frequently in women with PAPP-A concentrations below the 25th percentile, without, however, achieving statistical significance. Furthermore, a systematic review by Conde-Agudelo et al. that investigated the performance of PAPP-A with the prediction of PTB in twin pregnancies underlined its minimal predictive ability [30], revealing the possible inherent intricacy of the relationship between the risk of preterm delivery and PAPP-A concentrations.

### 4.2. Low PAPP-A and SGA/FGR

As already discussed, PAPP-A is essential in pregnancy for the regulation of placental function, fetal growth, and placental development through a molecular mechanism that entails the control of insulin-like growth factor activity, which affects multiple facets of pregnancy and fetal development. As noted by Queirós et al. [19] and confirmed by our meta-analysis, low PAPP-A levels pose an increased risk for the subsequent development of SGA/FGR, but this marker, by itself, possesses limited predictive value for identifying the bulk of at-risk cases, a finding that is in accordance with the predictive value of PAPP-A in singleton pregnancies, as demonstrated by Morris et al. [6]. Similar nonstatistical significant results were found in the studies by Saletra-Bielinska et al. [8] and Fox et al. [13], while Iskender et al. revealed an association between low PAPP-A levels and SGA/FGR, with this correlation approaching but not reaching statistical significance (*p* = 0.06) [12].

### 4.3. Low PAPP-A and Hypertensive Disorders of Pregnancy

The development of PE and hypertensive disorders of pregnancy, and their association with first-trimester biochemical markers has been thoroughly investigated [31,32], while the data remain conflicting in terms of twin pregnancies [33]. To begin with, not only have several studies failed to demonstrate an association between low PAPP-A levels and the subsequent development of hypertensive disorders of pregnancy [13,16], but some research groups have also underlined a prominent increase in the levels of PAPP-A in the affected pregnancies [7,14]. In singleton cases, PAPP-A levels are diminished during the first trimester, experiencing, however, a marked increase with the patient’s transition into the active phase of the disease. It is hypothesized that in twins, such an alteration occurs more swiftly, resulting in elevated levels earlier in pregnancy. A plausible explanation for such an observation could be placental over-compensation, as suggested by Svirsky et al., with the authors stating that this may happen if, in afflicted twin pregnancies, one placenta experiences decreased blood flow, prompting an increase in blood flow in the other placenta without adequate regulation [7]. As such, it is becoming clear by the results of the aforementioned studies and our meta-analysis that PAPP-A is a poor marker of the subsequent development of hypertensive disorders of pregnancy, and future studies should mostly focus on assays such as the soluble fms-like tyrosine kinase-1 (sFlt-1) and the placental growth factor (PLGF) [33].

### 4.4. Low PAPP-A and GDM

GDM is assumed to occur when the mother’s pancreas is unable to cope with the rising glucose load during pregnancy [15]. During normal pregnancy, β cells experience hyperplasia and hypertrophy to satisfy the metabolic requirements of the condition. Blood glucose levels increase when insulin resistance rises. Post-pregnancy, β cells, blood glucose levels, and insulin sensitivity revert to baseline levels. In gestational diabetes, β cells inadequately compensate for the increased demands of pregnancy, and when coupled with heightened insulin resistance, this leads to hyperglycemia. Post-pregnancy, β cells, blood glucose levels, and insulin sensitivity may normalize or may sustain impairment, potentially leading to gestational diabetes mellitus in future pregnancies or type 2 diabetes mellitus [34]. PAPP-A encodes a secreted metalloproteinase that cleaves insulin-like growth factor-binding protein (IGFBP), and it seems to play a part in controlling the bioavailability of IGF during pregnancy. This is really important as the IGF axis appears to be essential for both placental growth and function, as well as fetal growth, throughout pregnancy [12]. In singleton pregnancies afflicted by GDM, the initially diminished PAPP-A levels are subsequently markedly elevated alongside rising blood glucose levels throughout the active phase of the illness, whereas elevated PAPP-A levels in twin pregnancies are already observed in the first trimester. Consequently, there seems to be a rapid increase in PAPP-A levels in twin pregnancies that later manifest as GDM. This aligns with a greater burden of pregnancy in twin pregnancies compared to singleton pregnancies [15]. However, even increased levels of PAPP-A constitute a poor predictive marker of subsequent GDM, as depicted by Maymon et al., whose results demonstrated that integrating PAPP-A and maternal weight and employing logistic regression yielded a 55% detection rate for GDM with a 10% false-positive rate.

### 4.5. Low PAPP-A and Birth Weight Discordance

Regarding birth weight, previous studies have proven that low PAPP-A concentrations correlate with low birth weight in singleton pregnancies [35]. Research on first-trimester biomarkers in twin pregnancies is scarce. Saletra-Bielinska et al. conducted a retrospective analysis involving 304 individuals, which revealed no statistically significant relationships between PAPP-A concentrations and small for gestational age (SGA) or intertwin birth weight discordance (BWD) [8]. Iskender et al. investigated 104 twin pregnancies and did not identify any significant association between low levels of PAPP-A and unfavorable pregnancy outcomes; however, they noted a statistically nonsignificant tendency toward fetal growth disorders in patients with low PAPP-A levels [12]. There is one study specifically evaluating MCDA twins, which indicated a potential inclination for reduced PAPP-A MoM in pregnancies with a significant BWD when both twins were FGR (BW < 10th percentile) [18]. Despite the association between discordance in first-trimester markers and fetal growth discordance, as indicated by the studies mentioned above, the predictive value remains modest, and enhanced performance probably necessitates the incorporation of additional markers in a multimodal algorithm [18].

### 4.6. Low PAPP-A and Intrauterine Fetal Demise

The prenatal demise of one fetus during the second trimester in twin gestations presents a challenging dilemma for the obstetrician in terms of pregnancy management. The rarity of such a disorder and the lack of extensive studies hinder the ability to counsel parents regarding prognosis and best care, with several institutions implementing delayed-interval deliveries for the surviving twins, aiming to ameliorate the prognosis of those twins [36]. Nevertheless, the optimal management option is still under investigation, and recent research has focused on predicting such adversity. Intrauterine fetal demise has been linked to reduced levels of PAPP-A in singleton pregnancies [28]. Saletra et al. identified a strong correlation between higher PAPP-A levels and the risk of intrauterine death [8]. No prior linkage has been documented in twins; for instance, Fathian et al. examined unfavorable outcomes in twin pregnancies with PAPP-A levels beyond the 95th percentile and identified no relationships [37]. This is the very first report that indicated a greater incidence of IUD in women with elevated first-trimester PAPP-A levels. The proteolytic activity of PAPP-A on IGFBP suggests that increased levels may result in the reduced bioavailability of IGFBP. Wang et al. discovered markedly reduced levels of maternal serum IGFBP-3 in women delivering singletons before 32 weeks of gestation, likely attributable to elevated concentrations of PAPP-A [38]. The increased concentration of PAPP-A may similarly influence implantation and placentation as its decreased concentration. The desensitization of IGF receptors due to excessive IGF release may be a potential mechanism; however, additional investigations are required to validate this notion. This mechanism, which applies to preterm deliveries, might be associated with IUD in twin gestations with elevated PAPP-A concentrations.

### 4.7. Strengths and Limitations

To the best of our knowledge, this is the first meta-analysis to assess the possible association between the low levels of PAPP-A with the subsequent development of adverse perinatal outcomes. The main strength of the review lies in the fact that an extensive search strategy was applied in an effort to include all the available literature on this issue, with our review comprising a sample size based on a total of 11 studies (6 of which were also included in our meta-analysis) and 3741 twin pregnancies. Furthermore, the majority of the included studies were matched for BMI, a fact that minimizes the risk of the reported results being influenced by the presence of the aforementioned confounding factor, which is known to affect the levels of the adipokines under examination. The credibility of the evidence was also evaluated, pointing to its high quality.

We acknowledge that the present meta-analysis has several limitations. Several parameters may contribute to this. For instance, the methodological heterogeneity that was noted in these studies may result in significant selection bias that may prohibit clear conclusions. To begin with, six of the included studies are retrospective in nature and, except for one study, the population under study comprised both monochorionic and dichorionic twin pregnancies without a subanalysis based on chorionicity occurrence, possibly due to the small sample size of monochorionic cases. Furthermore, there is heterogeneity in the definition of hypertensive diseases since four different diagnostic modules were adopted across the included studies. Additionally, it should be emphasized that, in most of the studies, the presence of pre-existing hypertension was not documented, which could eventually impact the findings. The same issue applies to the diagnosis of GDM since two different diagnostic criteria were used. Moreover, the studies included in this review did not use the same cut-off values to define low PAPP-A, as other studies used the 10th centile of their measurements, one used the 25th centile, and one adopted values lower than 0.42 MoMs, while the rest did not specify their cut-off. Lastly, data regarding the nature of prematurity (iatrogenic or spontaneous) are lacking, and as such, the results of preterm delivery should be interpreted with caution.

## 5. Conclusions

Our study’s primary findings indicate that diminished PAPP-A levels are correlated with a heightened incidence of unfavorable perinatal outcomes in twin pregnancies, namely the occurrence of preterm delivery and the subsequent development of SGA/FGR. PAPP-A is frequently evaluated in first-trimester screenings for aneuploidies, and thus it would be economically advantageous as a predictive tool for assessing the potential development of adverse perinatal outcomes. Identifying women at elevated risk for such adversities in twin pregnancies may facilitate appropriate management and potential interventions. Furthermore, additional studies are required to elucidate the precise mechanism linking PAPP-A with all of the above-mentioned pathological conditions and assess the risk of pregnancy complications based on PAPP-A concentrations as a continuous variable.

## Figures and Tables

**Table 1:** Demographic characteristics of the included patients.

Year; Author	N	Monochorionicity	Age	BMI at Testing	Gestation Age at Time of Testing	Race (%) (White/Black/Asian/Other)	Spontaneous Conception (%)	Preexisting DM (%)	Preexisting Chronic Hypertension (%)	Nulliparous (%)	Smoking (%)	Previous PE or GH (%)	Gestational Age at Delivery
Laughon et al., 2009 [11]	70	n/a	36.1	n/a	n/a	72/4.2/6.9/6.9	n/a	n/a	n/a	n/a	4.3	n/a	36.4
Iskender * et al., 2013 [12]	10/94	0/4	29.8/30.3	n/a	n/a	n/a	n/a	n/a	n/a	100/90.4	0/4	n/a	n/a
Fox et al., 2015 [13]	340	n/a	36	23	n/a	80/n/a/n/a/n/a	24	n/a	n/a	n/a	n/a	n/a	n/a
Maymon ** et al., 2016 [14]	9/96	13	36/31	72 kg/65 kg	12.5(88d)/12.1(85d)	n/a	33/66	n/a	n/a	56/38	11/20	11/8.3	35/36.6
Svirsky et al., 2016 [7]	144	18	31	23.5	12.1	n/a	48	n/a	n/a	25	n/a	11	36 + 3
Maymon *** et al., 2018 [15]	11/134	1/n/a	32/31	82 kg/64 kg	12/12.1	n/a	64/64	n/a	n/a	36/41	18/18	n/a	36.8/36.4
Kim ** et al., 2019 [16]	35/497	n/a	33.8/32.8	22.59/21.74	n/a	n/a	4/47	n/a	n/a	n/a	n/a	n/a	35.14/36.43
Saletra-Bielinska * et al., 2020 [8]	31/245	16/108	33.51/34.12	23.7/22.97	n/a	n/a	93.5/81.22	n/a	n/a	n/a	9.7/5.71	n/a	32.65/35.12
Chen ** et al., 2020 [17]	86/683	21/165	32.5/31.6	22.85/21.6	11.9/11.98	n/a	32.5/34.3	n/a	n/a	88.3/87.7	n/a	n/a	n/a
Bang Loppke et al., 2023 [18]	762	762	n/a	n/a	n/a	n/a/1.6/2.8/4	93.3	n/a	n/a	45.2	8.1	n/a	n/a
Queiros et al., 2024 [19]	466	17.6	32.9	<20:12.2% 20–35: 51.7%	n/a	85.6/11.1/2.1/0.2	55.8	0.6	4,7	66.1	10.5	n/a	35.4

Abbreviations: BMI: body mass index, DM: diabetes mellitus, PE: pPre-eclampsia, GH: gestational Hypertensionhypertension. * Cases with PAPP-A < 10/cases with PAPP-A > 10. ** Cases with PE/cases without PE. *** Cases with GDM/cases unaffected.

**Table 2:** Methodological characteristics of the included studies.

Author, Year	Timing of Assessment (Weeks)	Type of Study	Study Center	Recruitment Period	Inclusion Criteria	Exclusion Criteria	Low PAPP-A Definition	Investigated Outcome
Laughon et al., 2009 [11]	11–14	Retrospective study	New York University Hospital, USA	2003–2004	Twin pregnancies CRL 45 mm to 84 mm First-trimester screening including PAPP-A and b-hCG Prenatal care in the NYU Maternal-Fetal Medicine group	Incomplete records Selective reduction Refusal to participate	≤25th centile	PTB < 32 weeks
Iskender et al., 2013 [12]	11–14	Retrospective study	Ankara, Turkey	2005–2011	Twin pregnancies CRL 45 mm to 84 mm First-trimester screening including PAPP-A and b-hCG	Incomplete records Refusal to participate	≤10th centile	PTB < 34 weeks PIH GDM Discordant fetal growth IUGR/SGA Selective IUGR
Fox et al., 2015 [13]	10–14	Retrospective study	New York, USA	2005–2013	Twin pregnancies that underwent first and second-trimester combined screening	Monochorionic twins Pregnancies with chromosomal abnormalities Refusal to participate	<0.42 MoM	PTB < 34 weeks PTB < 37 weeks HDP BW < 10th percentile IUD of at least one twin
Maymon et al., 2016 [14]	11–14	Prospective observational study	Tel-Aviv, Israel	2011–2013	Twin pregnancies CRL 45 mm to 84 mm First-trimester screening including PAPP-A and b-hCG Regular cycles CRL consistent with the date of fertilization in women used ART	Pregnancies with chromosomal, anatomic abnormalities or NT > 3.5 mm Selective reduction Maternal disease that could lead to fetal growth restriction Women already using aspirin or heparin Refusal to participate	Not defined	PE
Svirsky et al., 2016 [7]	11–14	Prospective observational study	Tel-Aviv, Israel	2011–2013	Twin pregnancies CRL 45 mm to 84 mm First-trimester screening including PAPP-A and b-hCG Regular cycles CRL consistent with the date of fertilization in women used ART	Pregnancies with chromosomal, anatomic abnormalities or NT > 3.5 mm Selective reduction Maternal disease that could lead to maternal hypertension or fetal growth restriction Women already using aspirin or heparin or IVF hormonal treatment Refusal to participate	Not defined	PE
Maymon et al., 2018 [15]	11–14	Prospective observational study	Zerifin, Israel	2011–2013	Twin pregnancies CRL 45 mm to 84 mm First-trimester screening including PAPP-A and b-hCG Regular cycles CRL consistent with the date of fertilization in women used ART	Pregnancies with chromosomal, anatomic abnormalities or NT > 3.5 mm Selective reduction Maternal cardiovascular disease or medication Refusal to participate	Not defined	GDM
Kim et al., 2019 [16]	10–14	Retrospective study	Seongnam, SouthKorea	2005–2017	Twin pregnancies that underwent first and second-trimester combined screening	Pregnancies with chromosomal or anatomic abnormalities Pregnancies that ended in termination, miscarriage, or fetal death before 20 weeks of gestation Pregnancies delivering small for gestational age neonates in the absence of PE Refusal to participate	Not defined	PE
Saletra Bielinska et al., 2020 [8]	10–14	Retrospective study	Warsaw, Poland	2013–2018	Twin diamniotic pregnancies CRL 45 mm to 84 mm First-trimester screening including PAPP-A and b-hCG	Incomplete records Pregnancies with chromosomal or anatomic abnormalities MCMA twins Complicated by TTTS Refusal to participate	≤10th centile	PTB < 32 weeks PTB < 34 weeks PTB < 37 weeks PIH/PE GDM Discordant fetal growth SGA IUD
Chen et al., 2020 [17]	11–14	Prospective observational study	Shanghai, China	2014–2017	Twin pregnancies CRL 45 mm to 84 mm First-trimester screening including PAPP-A and b-hCG	Incomplete records Pregnancies with chromosomal or anatomic abnormalities MCMA twins Selective reduction High risk for PE Women already using aspirin, heparin, or calcium Refusal to participate	Not defined	PE
Bang Loppke et al., 2023 [18]	11–14	Register-based national cohort study	Denmark	2008–2017	MCDA twins CRL 45 mm to 84 mm First-trimester screening including PAPP-A and b-hCG	Incomplete records Pregnancies with chromosomal or anatomic abnormalities Pregnancies underwent cord occlusion for other reason than sFGR Refusal to participate	Not defined	Growth discordance
Queiros et al., 2024 [19]	10–14	Retrospective study	Lisbon, Portugal	2010–2022	Twin pregnancies Delivery at ≥24 weeks	Incomplete records MCMA twins Pregnancies with chromosomal or anatomic abnormalities Single demise before 24 weeks Abnormal umbilical cords TORCH infections Complicated by TTTS/TAPS Refusal to participate	≤10th centile	PTB < 32 weeks PTB < 34 weeks PIH/Early and late onset PE SGA PTB associated with FGR and/or HDPs

Abbreviations: CRL: crown crown–rump length, PTB: preterm birth, PIH: pregnancy- induced hypertension, PE: pPre-eclampsia, GDM: gestation diabetes, SGA: small for gestational age, IUD: intrauterine demise, sFGR: selective fetal growth restriction, MCDA: monochorionic diamniotic, MCMA: mMonochorionic monoamniotic, NT: nuchal translucency, TTTS: twin- to- twin transfusion syndrome, ART: assisted reproductive technology, MoM: multiples of median, BW: birth weight, HDPs: hypertensive disorders of pregnancy.

**Table 3:** Summary of the outcomes of GDM, IUD, and discordant fetal growth and their association with PAPP-A in the included studies. Abbreviations: GDM: gestation diabetes, IUD: intrauterine demise, PAPP-A: pregnancy-associated plasma protein-A, NS: not studied.

Author, Year	GDM	IUD	Discordant Fetal Growth
Laughon et al., 2009 [11]	NS	NS	NS
Iskender et al., 2013 [12]	Low PAPP-A levels are not associated with GDM	NS	Low PAPP-A levels are not associated with discordant fetal growth
Fox et al., 2015 [13]	NS	Low PAPP-A levels are not associated with the IUD of at least one twin	NS
Maymon et al., 2016 [14]	NS	NS	NS
Svirsky et al., 2016 [7]	NS	NS	NS
Maymon et al., 2018 [15]	High levels of PAPP-A are associated with GDM	NS	NS
Kim et al., 2019 [16]	NS	NS	NS
Saletra Bielinska et al., 2020 [8]	Low PAPP-A levels are associated with GDM	Low PAPP-A levels are not associated with the IUD of at least one twin	Low PAPP-A levels are not associated with discordant fetal growth
Chen et al., 2020 [17]	NS	NS	NS
Bang Loppke et al., 2023 [18]	NS	NS	Low PAPP-A levels are not associated with discordant fetal growth
Queiros et al., 2024 [19]	NS	NS	NS
